# *BCL2L10/BECN1* modulates hepatoma cells autophagy by regulating PI3K/AKT signaling pathway

**DOI:** 10.18632/aging.101737

**Published:** 2019-01-26

**Authors:** Jiafa He, Li Deng, Heping Liu, Taiying Chen, Shengying Chen, Shangzhou Xia, Yubin Liu

**Affiliations:** 1 Department of Hepatobiliary Surgery, Guangdong General Hospital, Guangdong Academy of Medical Sciences, Guangzhou 510080, Guangdong, China; 2 Guangzhou Beogene Biotech CO., Ltd., Guangzhou 510000, Guangdong, China; 3 Guangzhou Yiyang Bio-technology Co., Ltd, Guangzhou 510000, Guangdong, China

**Keywords:** hepatocellular carcinoma, BCL2L10, autophagy, BECN1 (Beclin 1), PI3KC3

## Abstract

The aim of this study was to investigate *BCL2L10* and *BECN1* expression and their effect on autophagy in hepatocellular carcinoma (HCC). We found that *BCL2L10* expression was low in hepatoma tissues and cells. Overexpression of *BCL2L10* decreased the activity of hepatoma cells. To analyze autophagic flux, we monitored the formation of autophagic vesicles by fluorescence protein method. Autophagy-related protein LC3B-II was accumulated and P62 was decreased, which indicated that autophagy was induced by BECN1, while *BCL2L10* could suppress this trend. Immunofluorescence assay showed that *BCL2L10* and Beclin 1 were co-located in hepatoma cells. Immunoprecipitation showed that *BCL2L10* could inhibit the autophagy of hepatoma cells by combining with Beclin 1. ELISA and co-immunoprecipitation suggested that the combination between BCL2L10 and Beclin 1 reduced the bond between Beclin 1 and PI3KC3. Based on Kyoto Encyclopedia of Genes and Genomes (KEGG) pathway analysis, the PI3K/AKT signaling pathway was significantly upregulated in HCC. In conclusions, *BCL2L10* had a low expression in HCC tissues and cells, which could release *BECN1* to induce autophagy of hepatoma cells by downregulating PI3K/AKT signaling pathway.

## Introduction

With a 9.1% death rate globally in 2012, hepatocellular carcinoma (HCC) has become the second leading disease of cancer-related mortality [1]. In addition, HCC accounts for about 90% of primary liver cancers, which is a common malignant tumor and a significant cause of mortality in several regions of Africa and Asia. In 2008, half of the new liver cancer cases occurred in China throughout the world [2,3]. Furthermore, it is the fifth most common cancer and the third leading cause of cancer deaths globally [3]. Due to the lack of effective biomarkers for early diagnosis, most patients with HCC died within one year because of the late operation of radical resection, leaving patients with only radical treatment options including radiotherapy, interventional treatment, or chemotherapy [4].

The liver depends largely on the autophagy for pathology and physiology. Hence, autophagy is of vital significance for the pathogenesis of liver diseases and normal liver physiology [5]. Autophagy means the basic self-degradative physiological process of removing worn-out or damaged organelles and other cellular components, such as mitochondria, Endoplasmic Reticulum (ER), protein aggregates, peroxisomes and intracellular pathogens [6]. Being a key process in the cellular homeostasis and organelle turnover of every cell, autophagy can also promote cell death under certain stress conditions. Cancer cells require the autophagy so as to overcome certain stress conditions including nutrient deprivation and ROS (Reactive Oxygen Species) production via degradation of non-functional organelles and metabolites, as a result, inducing metabolite self-recruitment [7]. In particular, lysosome-mediated degradation is of pivotal importance in both stress responses and normal physiological conditions, such as in infection, carcinogenesis, proteotoxicity, and metabolic dysregulation. Therefore, related to a variety of liver diseases, the dysfunction of autophagy reveals a potential therapeutic approach [5,8].

*BECN1* (*Beclin 1*) is a central regulator of autophagy and a haplo-insufficient tumor suppressor that is decreased in many human tumors [1,9]. Involved in the degradative process of macroautophagy, BECN1 could bind to PI3KC3 to assemble autophagy-inducing complexes [1,9]. In addition, *BECN1* is a core component of the Vps15/p150 complex and Vps34/Class III PI3K (PI3KC3) that regulates multiple membrane trafficking events [9]. It has been recently found that the stress-induced Hsp70 could participate in macroautophagy by bounding with *BECN1*, which is a crucial component of the autophagy molecular complex [10]. *BECN1* as a tumor suppressor is also evidenced by the identification of their binding partners, most of which are implicated in tumorigenesis, such as BCL-2. The anti-apoptotic member of the *BCL-2* family constitutively binds to BECN1 and Ambra1 complex inhibiting autophagy induction [6].

There are at least 20 *BCL-2*-related proteins involved in the ‘life or death’ pathways of mammalian cells, among which *BCL2L10* is the most recently identified and the least studied [11]. *BCL2L10*, also known as *BCLB*, is widely expressed in human tissues, with the highest levels typically found in liver, pancreas, brain and lungs. Studies had proved that it was a methylated gene in hepatocellular carcinoma [11,12]. In particular, Bai et al. found a tumor-specific upregulation of *BCL2L10* in prostate, gastric, breast, non-small cell lung cancers and colorectal adenocarcinomas [13]. As for autophagy, BCL2L10 could bind to BECN1 that was an inducer of autophagy at BH1 or BH3 domain, reducing autophagic cell death in cervical cancer by mTor signaling pathway [26].

In this study, the expression of *BCL2L10* and *BECN1* and their effects on autophagy in HCC were observed. Then we further explored the negative relation between *BCL2L10* and *BECN1* as well as the autophagy-inhibitor that they played in HCC, which was consistent with a previously published work by Liu et al. [11], offering novel insights into the treatment of liver cancer. Furthermore, we conducted bioinformatics analysis to find the functional mechanism of *BCL2L10/ BECN1* in HCC, which indicated that regulation of autophagy and PI3K/AKT signaling pathways were inhibited by *BCL2L10/ BECN1* in HCC.

## RESULTS

### KEGG pathway enrichment analysis of DEGs

**Figure 1:**
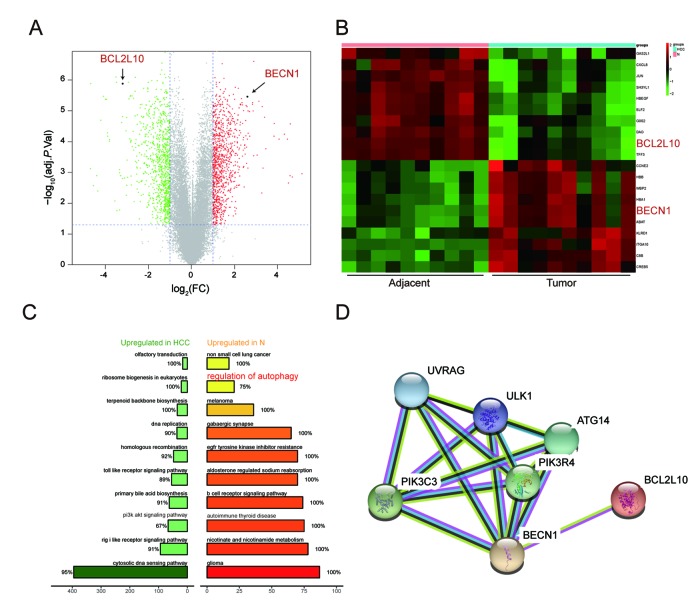
**Function annotations for *BCL2L10 /*** ***BECN1* in HCC.** (**A**-**B**) Hierarchical cluster analysis of the 10 most up and down regulated mRNAs. In the heat maps, green represents genes that are down-regulated whereas red represents genes that are up-regulated. (**C**) Plot of ten most enriched KEGG pathways in HCC. Pathways are ordered by normalized enrichment score (NES). Percentage beside the bar indicates the proportion of differential genes in pathway gene set. (**D**) STRING co-expression network for *BCL2L10/ BECN1* and their related signaling pathways.

**Figure 2:**
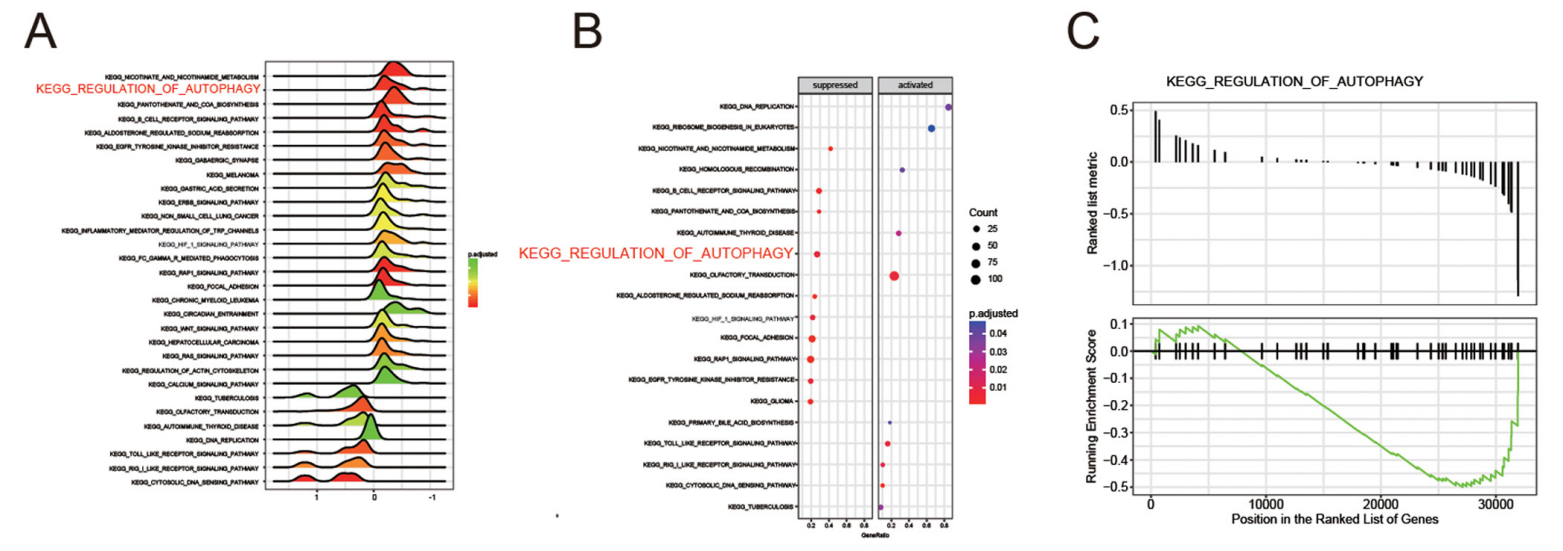
**The regulation of autophagy pathway was suppressed in HCC.** (**A**-**B**) Joyplot and dotplot suggested the distributions of some KEGG pathways gene sets in all differential genes. (**C**-**D**) Gseaplot showed the regulation of autophagy pathway was discovered in the region where genes were down-expressed in HCC.

All differential expression genes (DEGs) from GPL570 platform/ GSE49515 series were under the |log_2_FC| > 2 and adjusted *P* < 0.05 level of limma. Through differential genes expression analysis, expression of 1892 mRNAs showed significant difference where 947 were upregulated and 945 downregulated, including *BCL2L10* and *BECN1* (Figure 1A). The top 20 up- and down- regulated mRNAs were shown in Figure 1B. Then, we conducted KEGG pathway analysis based on HCC-related DEGs and obtained the 10 top and down scored pathways according to the enrichment scores from GSEA report (Figure 1C). Meanwhile, the consequence of STRING analysis on *BCL2L10* / *BECN1* related pathways were displayed in Figure 1D. After crosschecking those consequences, we narrowed down our interesting pathways into one mutual option, the regulation of autophagy pathway, which was suppressed in HCC. Thus, we speculated that hepatoma cell autophagy could be influenced by either *BCL2L10* or *BECN1*. Based on the fold change value of each pathway, the distribution of several KEGG pathways were drawn in joyplot and dotplot (Figure 2A-2B). To summarize, the autophagy pathway was upregulated in HCC (Figure 2C).

### The expression of *BCL2L10* was low but BECN1 was high at mRNA and protein level in HCC tissues and cells

**Figure 3:**
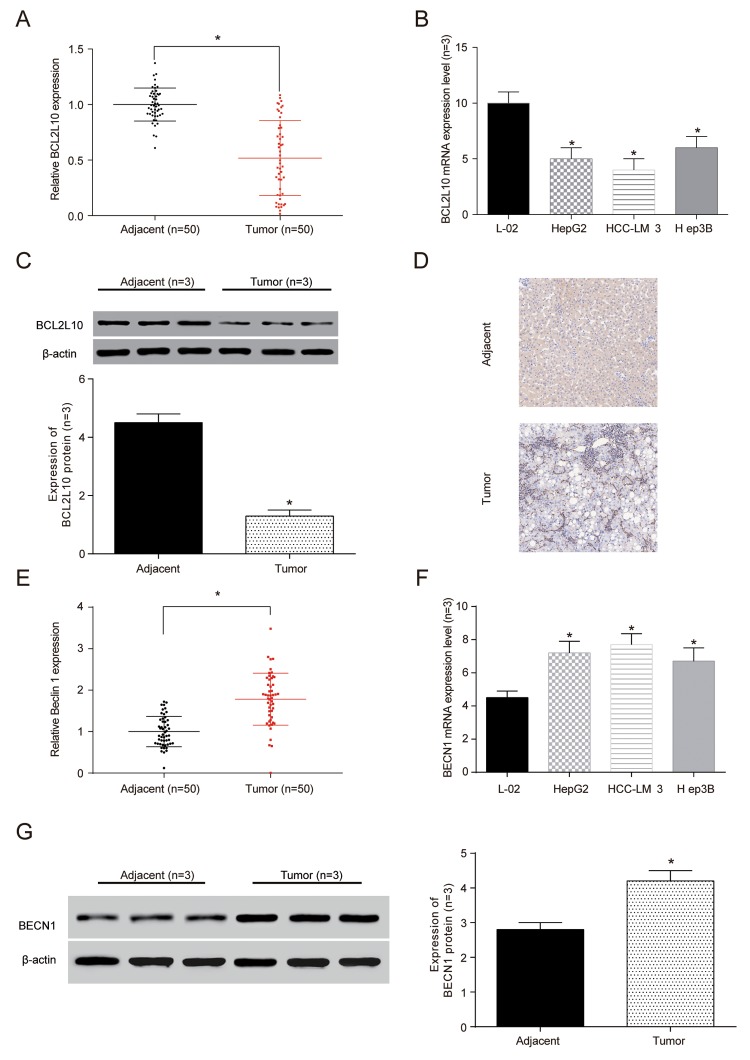
**The expression of** ***BCL2L10* was low in hepatocellular carcinoma (HCC) tissues and cells.** (**A**) The expression of *BCL2L10* mRNA in HCC detected by qRT-PCR. (**B**) The expression of *BCL2L10* in L-02 normal liver cells and 3 groups of hepatoma cells detected by qRT-PCR. (**C**) Western Blot showed the low expression of BCL2L10 in HCC tissues. (**D**) Immunohistochemistry showed that the normal liver tissues had more brown granules than HCC tissues (× 40). * *P*<0.05). (**E**) The expression of *BECN1* mRNA in L-02 normal liver cells and 3 groups of hepatoma cells detected by qRT-PCR. (**F**) The expression of *BECN1* in L-02 normal liver cells and 3 groups of hepatoma cells detected by qRT-PCR. (**G**) Western Blot showed the low expression of BECN1 in HCC tissues.

The expression of *BCL2L10* and *BECN1* in HCC tissues and cells were confirmed by qRT-PCR. The results exhibited that the expression of *BCL2L10* mRNA expression was lower in HCC tissues and cells compared with control groups (*P*<0.05, Figure 3A, 3B). Moreover, the BCL2L10 protein level in HCC tissues was significantly downregulated compared with normal liver tissues as well (*P*<0.05, Figure 3C) (× 40, Figure 3D). Similarly, we detected the expression of BECN1 to be upregulation in tumor tissues and HCC cells at mRNA level (*P* <0.05, Figure 3E, 3F) and protein level (*P* <0.05, Figure 3G).

### *BCL2L10* inhibited autophagy of HCC cells

**Figure 4:**
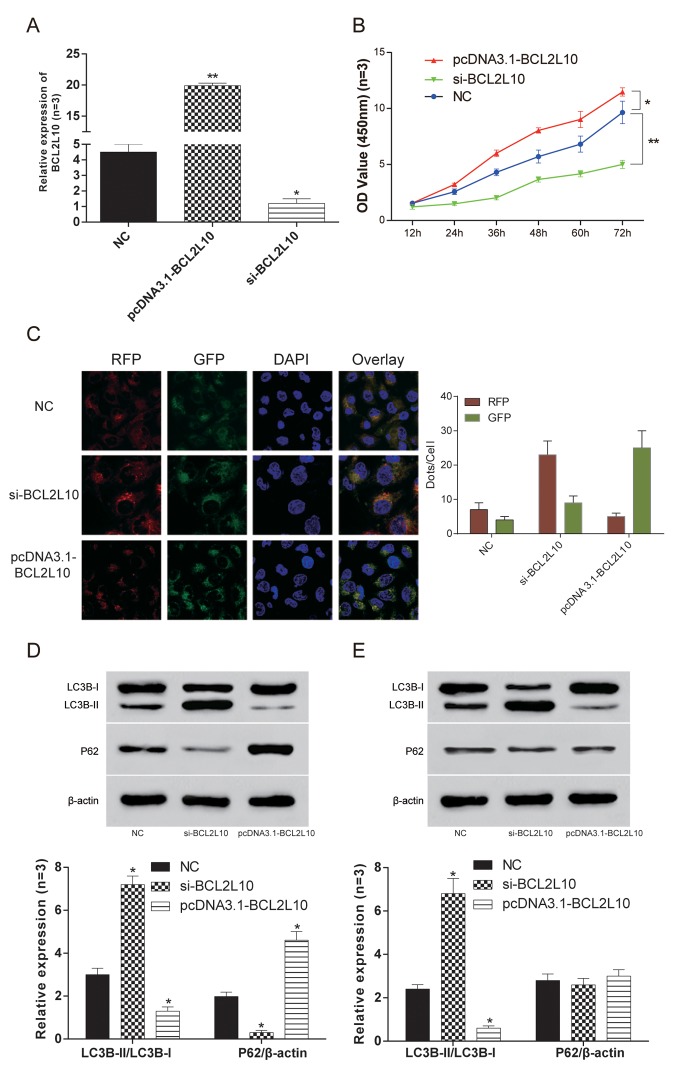
***BCL2L10* inhibited autophagy of Hep3B cells.** (**A**)The expression of *BCL2L10* in Hep3B cell line after transfection in three different groups was detected by qRT-PCR. (**B**) The cell viability was detected by CCK-8 in Hep3B cell line. The cell viability of si-*BCL2L10* group was the weakest, while the cell viability of pcDNA3.1-*BCL2L10* was the strongest. (**C**) The autophagy flux was observed under fluorescence microscope in Hep3B cell line. (**D**) The expression of LC3B-II/LC3B-I expression in the pcDNA3.1-*BCL2L10* group was decreased, while the expression of LC3B-II/LC3B-I in the si-*BCL2L10* group was increased. Besides, knockdown of *BCL2L10* inhibited autophagic P62 degradation. (**E**) Accumulation of LC3B-II was also observed in the presence of Bafilomycin A1, while P62 expression was little decreased in Hep3B cells with *BCL2L10* overexpression. * *P*<0.05, ** *P*<0.01.

Hep3B HCC cells with the highest expression level of *BCL2L10* were selected for the subsequent experiments. The expression level of *BCL2L10* in three different groups was verified by qRT-PCR (Figure 4A). Then loss-of-function and gain-of-function experiments were performed to detect the effect of BCL2L10 on autophagy, and the protein level of it was examined by western blot (Supplementary Figure 1A-B), as well as that of BECN1 (Supplementary Figure 1C-D). The cell viability of si-*BCL2L10* group was the weakest (*P*<0.05), while the cell viability of pcDNA3.1-*BCL2L10* group was the strongest (*P*<0.05) (Figure 4B). The autophagy flux was observed under fluorescence microscope (Figure 4C). Compared with NC group, the expression of LC3B-II/LC3B-I in pcDNA3.1-*BCL2L10* cells was decreased. In contrast, the expression of LC3B-II/LC3B-I in si-*BCL2L10* cells was increased, which indicated that the overexpression of *BCL2L10* suppressed the autophagy of hepatocarcinoma cells (*P*<0.05, Figure 4D). To test whether *BCL2L10* influenced autophagic flux, we also evaluated the expression of P62. Indeed, knockdown of *BCL2L10* inhibited autophagic P62 degradation (*P*<0.05, Figure 4D). Accumulation of LC3B-II was also observed in the presence of Bafilomycin A1 (an inhibitor of late-phase autophagy) (*P*<0.05, Figure 4E), while P62 expression was little decreased in Hep3B cells with *BCL2L10* overexpression (*P*>0.05, Figure 4E). *BCL2L10* overexpression failed to lead to decrease in P62 protein level in the presence of Bafilomycin A1, which indicates that autophagic degradation of P62 was blocked by Bafilomycin A1.

### *BCL2L10* and *BECN1* bind to each other in hepatocarcinoma cells

**Figure 5:**
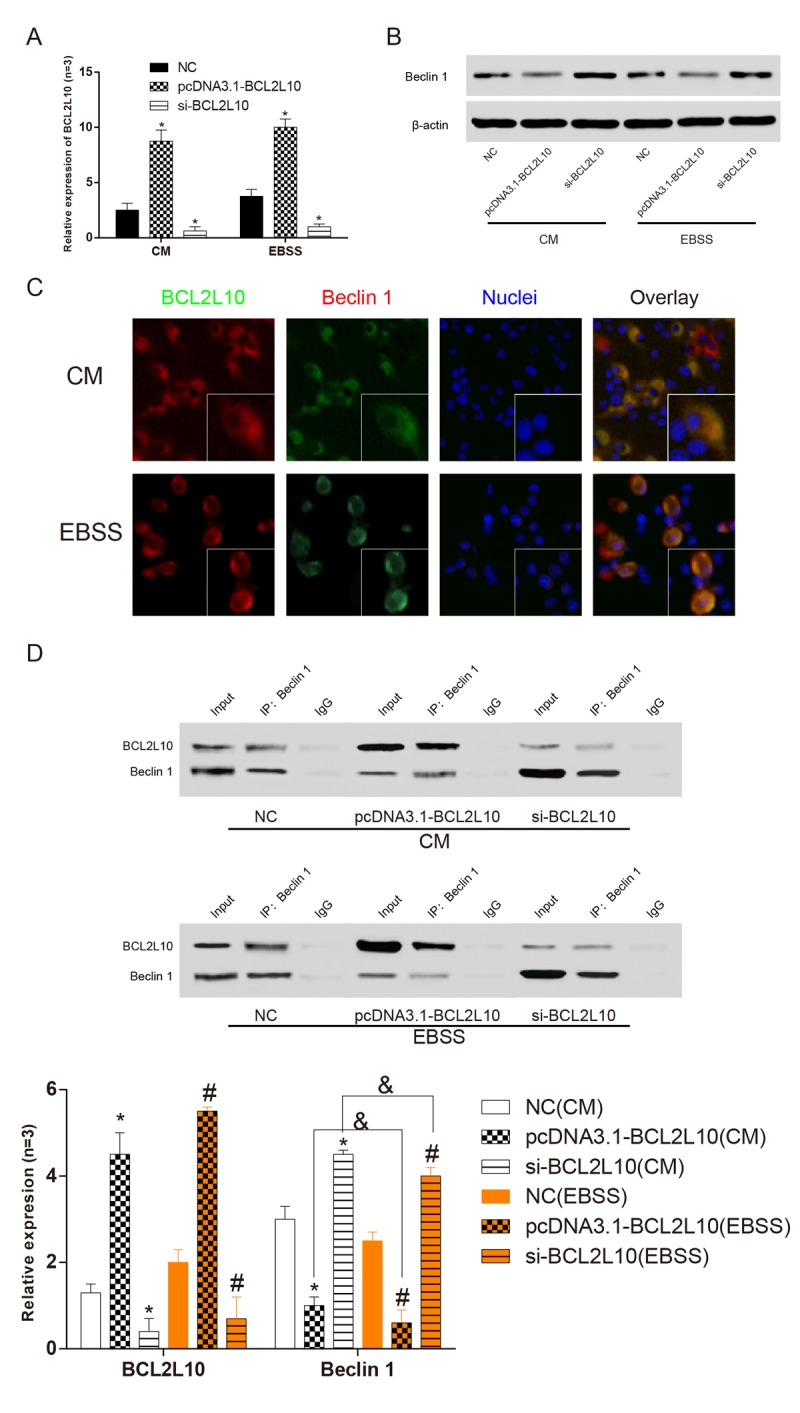
***BCL2L10* and *BECN1* bound to each other in** i**n Hep3B** **cells.** (**A**) qRT-PCR was used to detect the expression level of *BCL2L10* after transfection. (**B**) The expression level of Beclin 1 was detected by western blot. (**C**) The locations of *BCL2L10* and Beclin 1 in both complete medium and starved medium were observed by immunofluorescence. (**D**) The expression level of Beclin 1 in *BCL2L10* was detected by immunoprecipitation assay. CM: complete medium; EBSS: starvation medium. * means to compare with NC (CM) group *P*<0.05; # means to compare with NC (EBSS) group; & means EBSS groups compared with CM groups *P*<0.05.

The Hep3B hepatocellular carcinoma cells were cultured in CM normal medium and EBSS starvation medium for 6 hours and transfected with pcDNA3.1-*BCL2L10* or si-*BCL2L10* respectively. QRT-PCR (*P*<0.05, Figure 5A) and western blot (*P*<0.05, Figure 5B) were used to ascertain the expression levels of *BCL2L10* and *BECN1* (Beclin 1) in three different subgroups under two different culture conditions after transfection. The results showed that after the downregulation of *BCL2L10*, the transcription level of *BECN1* mRNA remarkably increased and the protein expression level of Beclin 1 also increased. The expression of *BECN1* changed conversely after overexpression of *BCL2L10*, which proved that the expression level of *BCL2L10* was negatively correlated with that of *BECN1*. To determine whether there is a direct interaction between *BCL2L10* and *BECN1*, we first detected the co-localization in HCC cells. The intracellular distribution of *BCL2L10* and *BECN1* was detected by immunofluorescence. The results (Figure 5C) showed that *BCL2L10* and Beclin 1 co-located in normal culture medium. This co-localization appeared commonly in the cytoplasm. The co-localization still existed when cells lacked nutrition, but the proportion of co-localization was small. The co-localization of the two proteins in the cells suggested that there may be a direct bounding between the two proteins. To further confirm this conclusion, we collected the above-mentioned cellular proteins for immunoprecipitation (*P*<0.05, Figure 5D). Beclin 1 was detected in the precipitated *BCL2L10* from CM and EBSS medium indicating that it bound to *BCL2L10* (*P*<0.05). In CM medium, the binding of *BECN1* and *BCL2L10* decreased after upregulating *BCL2L10*, and the autophagy was promoted. In the EBSS medium, this decreasing trend became more obvious (*P*<0.05). In CM medium, however, the binding of *BECN1* and *BCL2L10* increased after downregulating *BCL2L10*, and the autophagy was inhibited. In the EBSS medium, this increasing trend was suppressed (*P*>0.05). It further proved that *BCL2L10* could suppress autophagy by binding to *BECN1*, and this combination had a statistically significant difference in nutrient adequacy and nutrient deficiency.

### The BECN1 BH3 domain and BCL2L10 domain BH1 were required for BCL2L10-BECN1 complex

It was reported that the mutation in the BH3 domain of BECN1 and BH1 domain of BCL2L10 both blocked the interaction of BCL2L10 with BECN1 [14,15]. Thus, we conducted co-immunoprecipitation to verify this result in HCC cells transfected with BCL2L10-WT (wide type) and BCL2L10ΔBH1. We found that the binding of BCL2L10ΔBH1 to BECN1 was markedly reduced, suggesting that BH1 domain on BCL2L10 was necessary for this binding (Supplementary Figure 1F). Similar, we transfected BECN1-WT (wide type) and BECN1ΔBH3 into HCC cells and witnessed BECN1ΔBH3 failed to co-immunoprecipitate with BCL2L10, which confirmed BH3 domain on BECN1 was essential to bind to BCL2L10 (Supplementary Figure 1E).

### Overexpression of *BCL2L10* inhibited autophagy of hepatocarcinoma cells by binding to *BECN1*

**Figure 6:**
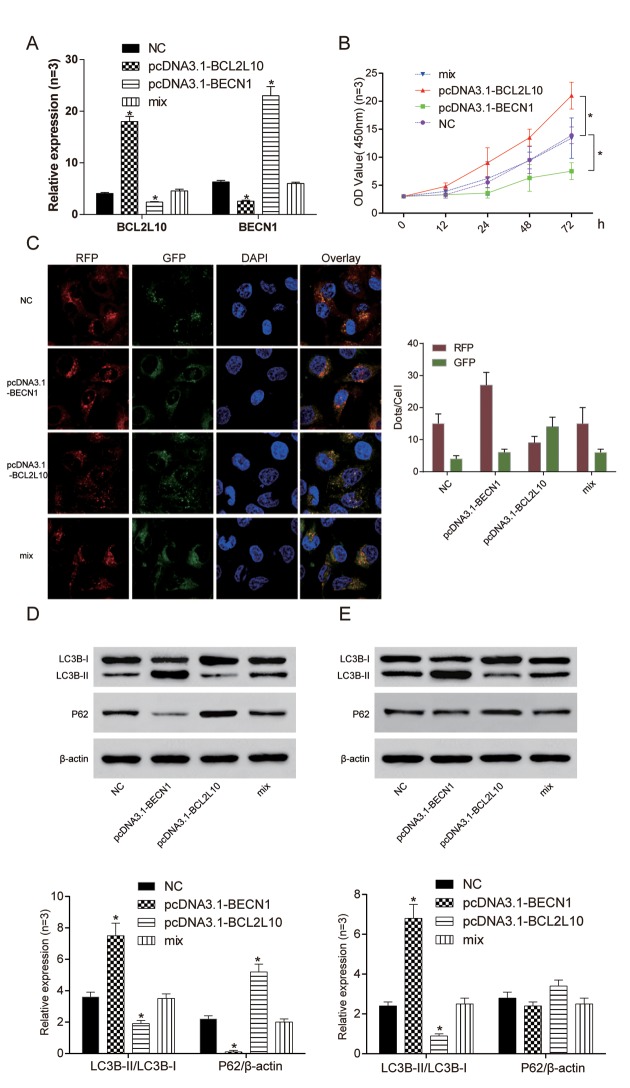
**Overexpression of *BCL2L10* inhibited autophagy of Hep3B cells by binding to *BECN1.*** (**A**) The expression of *BCL2L10* and *BECN1* after transfection in four different groups was detected by qRT-PCR. (**B**) The cell viability of pcDNA3.1-*BECN1* group was the weakest, while the cell viability of pcDNA3.1-*BCL2L10* group was the strongest. (**C**) Overexpression of *BECN1* facilitated the accumulation of LC3B puncta in Hep3B cells as detected by immunofluorescence. (**D**) The expression of LC3B-II/LC3B-I in pcDNA3.1-*BCL2L10* group was decreased while the expression of LC3B-II/LC3B-I in pcDNA3.1-*BECN1* group was increased. The changes of P62 had a contrary trend with changes of LC3B-II/LC3B-I. (**E**) Degradation of P62 was little observed in the presence of Bafilomycin A1 (an inhibitor of late-phase autophagy), while LC3B-II/LC3B-I expression was increased in Hep3B cells. * *P* <0.05.

In order to study *BECN1* when *BCL2L10* inhibited the autophagy of hepatocarcinoma cells, we transfected pcDNA3.1-*BECN1* and pcDNA3.1-*BCL2L10* into Hep3B cell line. The expression levels of *BCL2L10* and *BECN1* in four different groups were verified by qRT-PCR (Figure 6A). The cell viability of pcDNA3.1-*BECN1* group was the weakest (*P*<0.05), and the cell viability of pcDNA3.1-*BCL2L10* group was the strongest (*P*<0.05, Figure 6B). Moreover, overexpression of *BECN1* indicated autophagy induction in HCC cells as detected by immunofluorescence (Figure 6C). The expression of LC3B-II/I and P62 were detected with the absence of Bafilomycin A1 by Western Blot. The expression of LC3B-II/LC3B-I in pcDNA3.1-*BCL2L10* cells was decreased compared with NC group (*P*<0.05). On the contrary, the expression of LC3B-II/LC3B-I in the pcDNA3.1-*BECN1* group was increased (*P*<0.05). There was no significant difference in the expression of LC3B-II/LC3B-I between the NC group and mix group (*P*>0.05). The changes of P62 had a contrary trend with changes of LC3B-II/LC3B-I (*P*<0.05, Figure 6D). Little degradation of P62 was observed in the presence of Bafilomycin A1 (an inhibitor of late-phase autophagy) (*P*<0.05, Figure 6E), while LC3B-II expression was decreased in Hep3B cells with pcDNA3.1-*BCL2L10* transfection (*P*>0.05, Figure 6E). It suggested that the overexpression of *BCL2L10* inhibited autophagy of hepatocarcinoma cells by downregulating *BECN1*, whereas overexpression of *BECN1* prevented *BCL2L10* from inhibiting autophagy.

### *BCL2L10* defect induced autophagy of hepatocarcinoma cells by releasing *BECN1*

**Figure 7:**
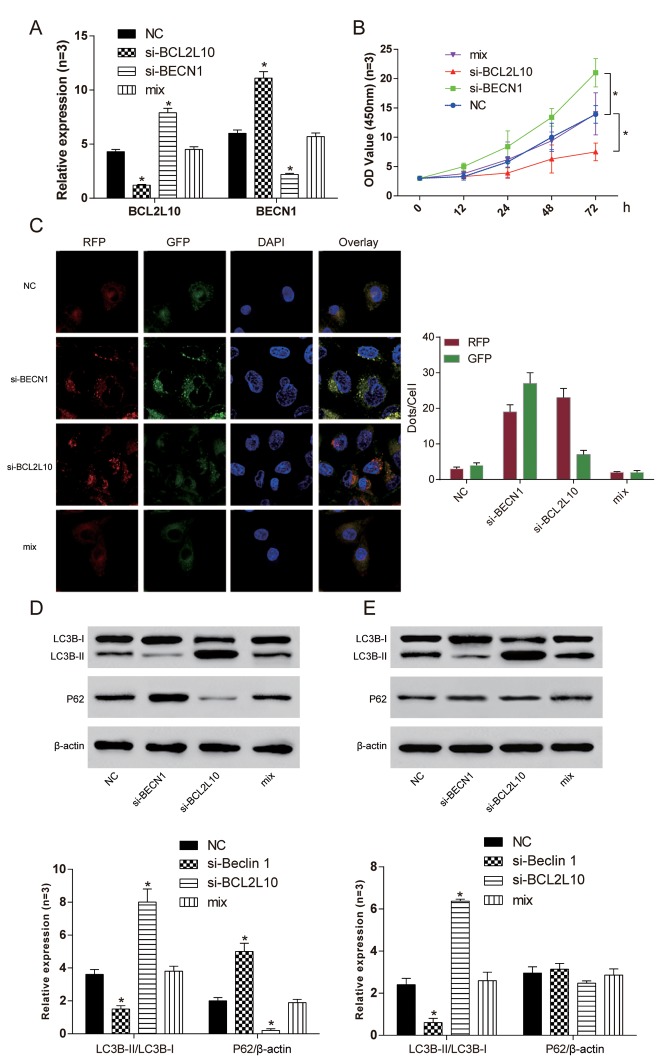
**Si-*BCL2L10* induced autophagy of Hep3B** **cells by releasing *BECN1.*** (**A**) The expression of *BCL2L10* and *BECN1* after transfection in four different groups was detected by qRT-PCR. (**B**) The cell viability of si-BCL2L10 group was the weakest, while the cell viability of si-BECN1 group was the strongest. (**C**) Decrease of BECN1 could inhibit autophagic flux in HCC cells according to mRFP-GFP-LC3 assay. (**D**) The expression of LC3B-II/LC3B-I in si-*BECN1* group was decreased while the expression of LC3B-II/LC3B-I in si-*BCL2L10* group was increased. (**E**) Accumulation of LC3B-II was observed in the presence of Bafilomycin A1 in Hep3B cells. At the same time, knockout of *BECN1* could not induced autophagic P62 degradation. * *P* <0.05.

The expression levels of *BCL2L10* and *BECN1* in four different groups were verified by qRT-PCR (Figure 7A). The cell viability of si-*BCL2L10* group was the weakest (*P*<0.05), and the cell viability of si-*BECN1* group was the strongest (*P*<0.05, Figure 7B). We further found that si-*BCL2L10* could induce autophagic flux in HCC cells according to mRFP-GFP-LC3 assay (Figure 7C). Similarly, the influence of BCL2L10ΔBH1 on autophagic flux was detected to be increased (Supplementary Figure 2A). The expression of LC3B-II/I and P62 was detected by Western Blot. The expression of LC3B-II/LC3B-I in si-*BECN1* cells was decreased compared with NC group. On the contrary, the expression of LC3B-II/LC3B-I in the si-*BCL2L10* group was increased. There was no significant difference in the expression of LC3B-II/LC3B-I between the NC group and mix group (*P* <0.05, Figure 7D). Accumulation of LC3B-II was observed in the presence of Bafilomycin A1 in Hep3B cells (*P*<0.05). At the same time, neither si-*BCL2L10* nor si-*BECN1* could induce autophagic P62 degradation (*P*>0.05, Figure 7E). The results revealed that the *BCL2L10* defect induced autophagy of hepatocarcinoma cells by releasing *BECN1*.

### The combination of *BCL2L10* and *BECN1* reduced the bonding of *BECN1* and PI3KC3

**Figure 8:**
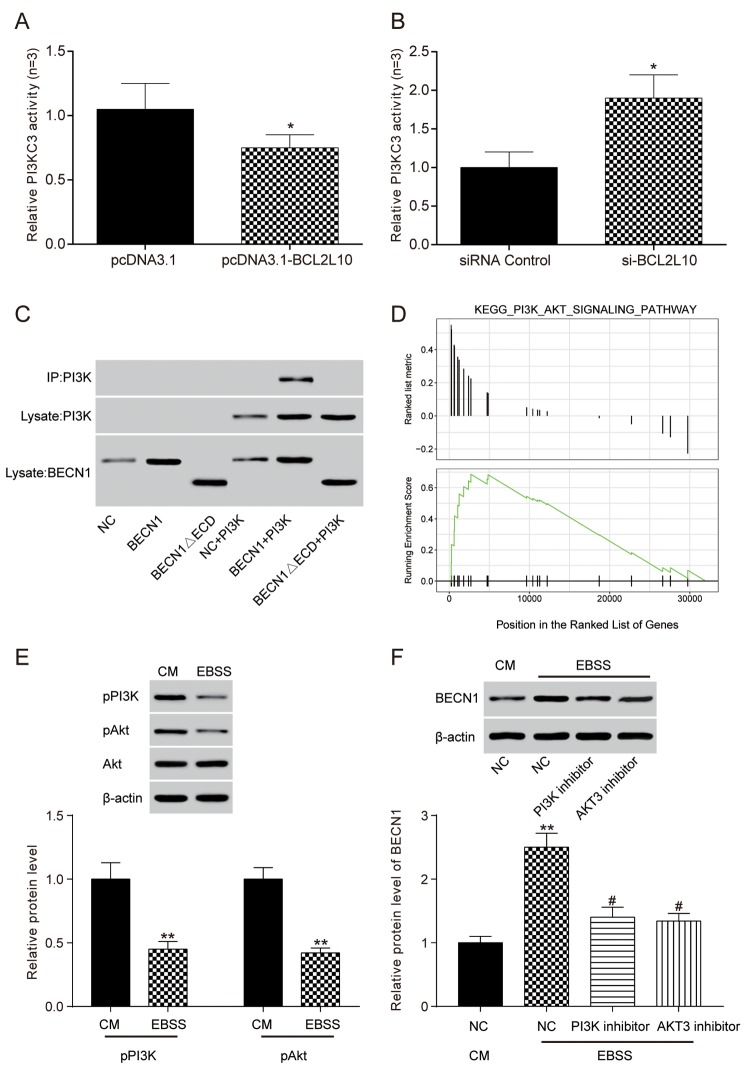
**The combination of *BCL2L10* and Beclin 1 reduced the bonding of Beclin 1 and PI3KC3 in Hep3B cell line.** (**A**) The activity of PI3KC3 after the overexpression of *BCL2L10* was detected by ELISA; (**B**) The activity of PI3KC3 after inhibiting *BCL2L10* was detected by ELISA; (**C**) The binding relation between PI3KC3 and BECN1 was detected by co-immunoprecipitation. (**D**) Bioinformatics turned out that PI3K/Akt signaling pathway was enabled in HCC. (**E**) PI3K p110α and p-AKT protein level was detected by Western blot after autophagy induction. (**F**) The BECN1 protein level was detected by western blot after adding PI3K inhibitor and AKT inhibitor. * *P*<0.05 ** *P*<0.01 compared with CM/NC group, # *P*<0.05 compared with EBSS/NC group.

ELISA was adopted to detect compounds related to *BECN1*. After the overexpression of *BCL2L10*, the relative activity of PI3KC3 kinase was decreased (*P*<0.05, Figure 8A), and the relative activity of PI3KC3 kinase was increased after inhibiting the expression of *BCL2L10* (*P*<0.05, Figure 8B). The above results demonstrated that *BCL2L10* could reduce the binding between Beclin 1 and PI3KC3 kinase. To further confirm the BCL2L10**-**BECN1-PI3KC3 complex in autophagy, we performed co-immunoprecipitation assay using BECN1ΔECD that was reported to destroy the BECN1-PI3KC3 complex by Furuya et al. [16]. The result illustrated that PI3KC3**/**Vps34 lost its ability to bind to BECN1ΔECD, indicating the importance of the BECN1 ECD domain for its interaction with PI3KC3 (Figure 8C).

### *BCL2L10/BECN1* inhibited hepatoma cell autophagy by upregulating PI3K/AKT signaling pathway

A previous study investigated the function of the protein BCL2L10 in gastric carcinoma through PI3K-Akt signaling pathway [17]. In our study, we conducted bioinformatics analysis, finding that based on KEGG pathway analysis, the PI3K/AKT signaling pathway was upregulated in HCC (Figure 8D). After incubated in EBSS starvation medium to induce autophagy, HCC cells were lysed to detect p-PI3K and p-AKT expression by western blot, which was decreased, showing the blockage of PI3K/AKT signaling pathway in autophagy (Figure 8E)**.** On the other hand, HCC cells were incubated in complete medium supplemented with 3-methyladenine (3-MA, an autophagy inhibitor), then added PI3K inhibitor (LY294002) and AKT inhibitor (MK2206). BECN1 level was detected to be upregulated by western blot (Figure 8F) and the autophagic flux was increased (Supplementary Figure 2A), suggesting that blockage of PI3K/AKT signaling pathway could induce autophagy. In short, BCL2L10 could inhibit autophagy by binding to BECN1 through PI3K/AKT signaling pathway activation (Supplementary Figure 2B).

## DISCUSSION

HCC resulted in high death rate worldwide. Currently, even with multidisciplinary comprehensive treatment, this tumor disease still has a poor prognosis and the recurrence rate is >50% [18]. Therapy for HCC, therefore, is still inefficacious [7]. Patients with hepatitis B or C, even without development of cirrhosis, face a high risk of HCC. Chemoprevention by phytochemicals with potent antioxidant and anti-inflammatory properties represents a fascinating strategy, which recently has been a subject of intense investigation [19]. The treatment and prognosis of HCC mainly depends on numerous factors, especially tumor stage and size [19].

Studies have proved the importance of apoptosis in therapeutic tumor-cell death, but many notable studies have confirmed that other forms of cell death are crucial especially when dealing with the whole tumor instead of isolated tumor cells [3]. In cancer cells, activation autophagy is considered as a cytoprotective factor [20]. It has been found to be vital in tumor suppression and previous studies have indicated the autophagy as a promising target for cancer therapy [21,22]. In various tumor cells, several chemotherapeutic agents have been observed for inducing autophagic cell death. Hu et al. investigated the potential of AZD8055 as a therapeutic agent in HCC partially through the perspective of inducing autophagic cell death [23]. Study of Chen et al. demonstrated lapatinib-induced cytotoxicity of HCC through the mechanisms of autophagic cell death [24].

*BCL2L10* is an apoptosis-related member of the *BCL-2* protein family. It’s also an anti-apoptotic oocyte-inherited 311 transcript and elimination of *BCL2L10* accelerates oocyte death [25]. The fate of a cell is largely mediated by the *BCL-2* family which has either pro- or anti-apoptotic activities [13]. Liu et al*.* found that BCLB protein expression was significantly correlated with low tumor stage and *BCLB* methylation is a frequent cancer-specific phenomenon. They further proved that *BCLB* acts as a tumor suppressor in HCC [11]. The autophagy-inducing effect of BCL2 family protein in HCC cells has also been reported [11]. Through qRT-PCR and Western Blot, we found that the expression of *BCL2L10* mRNA was low in HCC tissues and cells, which proved that compared with normal liver tissue, the expression of *BCL2L10* protein in HCC was also noticeably downregulated. We also observed that *BCL2L10* inhibited autophagy of HCC cells. Compared with NC group, the expression of LC3B-II in pcDNA3.1-BCL2L10 cells was decreased, contrarily, the expression of LC3B-II in si-*BCL2L10* cells was upregulated, which indicated that the overexpression of *BCL2L10* inhibited the autophagy of hepatocarcinoma cells, while si-BCL2L10 induced autophagy of hepatocarcinoma cells. The conflict about autophagy might result from the different underlying mechanism.

Approximately 30 specific genes that regulate autophagy have been identified in yeast, with 16 homologues in human. Among these genes, *BECN1* plays a key role in mammalian autophagy [26]. *BECN1* (also called *Beclin 1*) is essential for double membrane autophagosome formation, which is a vital step in autophagic nucleation. Abnormal expression of *BECN1* has been found in human melanoma, colon, ovarian and brain cancers [27]. In Al-Shenawy’s study, *BECN1* expression was significantly lower in the larger tumors. This indicated that decreased *BECN1* expression in HCC might contribute to the autophagy defect, which would affect the clinical prognosis. Likewise, Furuya *et al.* discovered that a mutant domain (ECD) on *BECN1* led to the ruin of *BECN1-*PI3KC3 complex, inhibiting autophagy [16]. Thus, we employed BECN1ΔECD to verify this point. Al-Shenawy proposed a hypothesis that a downregulated autophagy can contribute to tumor progression [28]. Our study had found that *BCL2L10* and *BECN1* bound to each other in hepatocarcinoma cells. It was observed that *BCL2L10* could reduce autophagy by binding to *BECN1*, and this combination had a statistically significant difference in nutrient adequacy. We also proved that overexpression of *BCL2L10* inhibited autophagy of hepatocarcinoma cells by binding to *BECN1*. It has been well-demonstrated by Robert *et al.* that BCL2 family protein such as BCL2L10 could bind to BECN1 to inhibit autophagy in cervical cancer cells by mTOR upregulation [29]. Intriguingly, mTOR was involved in the PI3K/AKT signaling pathway in autophagy [30]. Thus, we hypothesized that BCL2L10 suppressed autophagy by binding to BECN1 through PI3K/AKT signaling pathway activation in HCC cells, and finally validated it. Despite of the different cancer types, the mechanism still worked. In addition, we discovered *si-BCL2L10* induced autophagy of HCC cells by releasing *BECN1*.

Phosphatidylinositol 3-phosphate (PI3P) was reported to be essential in vesicular trafficking, organelle biogenesis and autophagy [31]. Phosphoinositide 3-kinase (PI3K) family members participate in various cellular fates, such as proliferation, survival, and cell growth [32]. The PI3K pathway, a critical signal transduction system linking oncogenes and multiple receptor classes to many essential cellular functions, is perhaps the most commonly activated signaling pathway in human cancer. PI3K-III is an important regulator of autophagy, a cellular response to nutrient starvation [2,9]. *BECN1*-class III phosphatidylinositol 3-kinase (PI3KC3) can produces an autophagy-specific pool of phosphatidylinositol 3-phosphate (PI3P). Inhibiting the activity or lacking lipid kinase components would suppress autophagy [33]. In this study, we noticed that the combination of *BCL2L10* and *BECN1* destroyed the bonding of *BECN1* and PI3KC3.

There have been resemblances to some extent between the current work and a previously published work by Liu et al. [11]. We performed bioinformatics analyses of pre-existing global gene expression data from PBMCs of healthy patients, eventually identifying differential expression of *BCL2L10*. Liu et al. used a more direct approach with their own primary data from various HCC lines before and after treatment with chemotherapeutic agents, also leading to the identification of *BCL2L10*. However, it's important to note that our studies are certainly more different than theirs. For example, we have conducted a series of *in vitro* experiments to reveal the combination between *BCL2L10* and *BECN1* and their effect on PI3K/AKT signaling pathway in HCC autophagy. On the other hand, Liu et al. suggested that *BCLB* expression was a starvation stress sensor inducing apoptosis and autophagy simultaneously in HCC cells through the adenosine monophosphate-activated protein kinase AMPK-mTOR signaling cascade. Taken together, we may draw an inference that there may exist possible relationship between the PI3K/Akt and AMPK/mTOR axes in HCC autophagy [34], which should be the next frontier of study.

To summarize, in this study, the expression of *BCL2L10* and *BECN1* in hepatoma cells and their effects on the autophagy of hepatoma cells was observed. *BCL2L10* had a low expression in HCC tissues and cells, which could combine with *BECN1* to induce autophagy of hepatoma cells. PI3K/AKT signaling pathway was involved in the regulation of autophagy by *BCL2L10/ BECN1*, which could be safely concluded from our bioinformatics analysis. A limitation of this study was that we only researched one cell line. Hence, there is a possibility that other elements could affect the results of our study. Further examinations should be needed to clarify the roles of *BCL2L10* and *BECN1* in HCC by means of including more cell lines such as HepG2 and HCC-LM3. Though our results may be impacted by the small sample size, they can still provide impactful ideas for the development of liver cancer therapy.

## METHODS

### Research object

In this study, we collected 50 groups of HCC tissues and their corresponding adjacent tissues from Guangdong General Hospital, Guangdong Academy of Medical Sciences (Supplementary Table 1). All tissues were collected from patients who had not received any form of chemotherapy or radiotherapy before surgery. All experiments were conducted with patients’ consent and approved by the Ethics Committee of Guangdong General Hospital, Guangdong Academy of Medical Sciences. HepG2 (ATCC, Manassas, VA, USA), HCC-LM3 (BNCC, Shanghai, China) and Hep3B (ATCC) were cultured in 90% high glucose DMEM (Invitrogen, Carlsbad, CA, USA) + 10% FBS (Invitrogen). Normal hepatocyte line L-02 (HL-7702, BNCC) was cultured in 90% RPMI-1640 (# 31800022, GIBCO, Grand Island, NY, USA) + 10% FBS (Invitrogen).

### GEO analysis

We downloaded gene expression data from GEO (https://www.ncbi.nlm.nih.gov/geo/query/acc.cgi). Peripheral blood mononuclear cell (PBMC) from 10 patients with HCC and 10 healthy individuals were isolated and total RNA was extracted for Affymetrix gene microarray analysis (Supplementary Table 2). The genes with differential expression were screened using the Bayesian test and fold change (*P*<0.05, and | log_2_(fold change) |> 1).

### MRNAs processing by STRING and GSEA

The Search Tool for the Retrieval of Interacting Genes database (STRING) is a pre-computed global resource for functional association prediction between genes. In this study, the STRING online tool was applied to analyze HCC-related genes with dysregulated expression, *BCL2L10* and *BECN1*. The outcome of STRING analysis would provide us with the GO term and KEGG pathways linked to the entered genes. Afterwards, the expression data of total normalized mRNAs were uploaded to GSEA v3.0 software. KEGG pathway gene set was used to conduct gene set enrichment analysis. Default weighted enrichment statistic was adapted to process data for 1000 times with normalization. *P*<0.05 was considered to be significantly enriched. Next, the 10 most significantly upregulated and 10 most significantly downregulated results of GSEA reports were selected to undergo graphics processing using “ggplot2” package in R language. In addition, we employed R language “GSEABase” package to handle the data. The gene sets represented by more than 10 and less than 500 genes were retained. The *P* values were adjusted by the Benjamini-Hochberg Procedure. A permutation test with 1000 times was utilized to identify the significantly differential pathways with *P*<0.05. We adopted “joyplot” and “dotplot” function to visualize their distribution.

### Mutagenesis and plasmid construction

Flag-BECN1ΔBH3 and Myc-BCL2L10ΔBH1 were obtained according to Robert et al. [29]. Flag-BECN1ΔECD was generated by two-step PCR mutagenesis. All of them were clone into pTRE vector (BD Bioscience Clontech, Palo Alto, CA, USA) as described previously [35].

### Cell culture and transfection

Hep3B cells were divided into two groups: (1) cultured in complete medium (90% DMEM + 10% FBS, Invitrogen) at 37 °C in a humidified incubator with 5% CO_2_; (2) cultured in Earle's Balanced Salt Solution (EBSS) starvation medium (GIBCO, # 14155063) at 37 °C in a humidified incubator with 5% CO_2_. HepG2 and HCC-LM3 cells were cultured in CM1-1 medium (90% DMEM-H + 10% FBS) at 37 °C in a humidified incubator with 5% CO_2_. They were digested and passaged with 0.25% trypsin (Invitrogen) when the cell confluence reached 90%. Transfection of plasmid, mutant genes and siRNA was carried out separately in accordance with the transfection instructions of Lipofectamine 3000 (Invitrogen, Cat: 12566014), and the final concentrations of plasmid and siRNA were 1 μg/mL and 25 nM, respectively. After transfection for 24 h, the transfection efficiency of cells was estimated by qRT-PCR. Cell transfection experiment was split up into three groups: (1) NC group: transfecting empty plasmid vector pcDNA3.1 (Invitrogen, # V79020) and siRNA control (5'-UUCUCCGAACGUGUCACGU-3') into hepatocarcinoma cells; (2) pcDNA3.1-*BCL2L10* group: transfecting pcDNA3.1-*BCL2L10* and siRNA into hepatocarcinoma cells; (3) si-*BCL2L10* group: transfecting empty plasmid vector pcDNA3.1 and si-*BCL2L10* (5'-AUGGCUCUUCCUUGAGUGAAA-3') into hepatocarcinoma cells. The other transfection experiment wass split up into four groups: (1) NC group: transfecting empty plasmid vector pcDNA3.1 into hepatocarcinoma cells (2) pcDNA3.1-*BCL2L10* group: transfecting pcDNA3.1-*BCL2L10* into hepatocarcinoma cells; (3) pcDNA3.1-*BECN1* group: transfecting pcDNA3.1-*BCL2L10* into hepatocarcinoma cells; (4) pcDNA3.1-*BCL2L10* and pcDNA3.1-*BECN1* group: transfecting pcDNA3.1-*BCL2L10* and pcDNA3.1-*BECN1* into hepatocarcinoma cells. Another transfection was divided into four groups: (1) NC group: transfecting siRNA control into hepatocarcinoma cells; (2) si-*BCL2L10* group: transfecting si-*BCL2L10* into hepatocarcinoma cells; (3) si-*BECN1* group: transfecting si-*BECN1* into hepatocarcinoma cells; (4) si-*BCL2L10* and si-*BECN1* group: transfecting si-*BCL2L10* and si-*BECN1* into hepatocarcinoma cells.

### qRT-PCR

For the qRT- PCR analysis of mRNAs, total RNAs were isolated from harvested cells using Trizol reagent (Invitrogen, Cat: 15596026), followed by further purification with RNeasy Mini kit and RNase-free DNase Set (Qiagen, Shanghai, China) in accordance with the instructions. To determine the mRNA levels in hepatoma cell lines, total RNAs were treated with DNase I (AM1906; Ambion, Austin, TX, USA) to eliminate genomic DNA contamination, and then reversely transcribed using Advantage RT-4PCR kit (Clontech, Mountain View, CA, USA). In order to quantify the relative expression levels of RNA, the $2^{-∆∆C_{t}}$ method was employed. Primer sequences used were given in Table 1 and GAPDH was considered as the internal reference. All the plasmids related to *BECN1* were synthesized by Shanghai GenePharma Co., Ltd (Shanghai, China).

**Table 1:** Primer sequences.

**Primer**	**Sequence (5’-3’)**
**BCL2L10-F**	5’- AATATATTGGGGGTTGGGGGTT -3’
**BCL2L10-R** **BECN1-F** **BECN1-R**	5’- AACAACAACTCAATACACTCCCA -3’ 5’- AGGAACTCACAGCTCCATTAC -3’ 5’- AATGGCTCCTCTCCTGAGTT -3’
**GAPDH-F**	5’- GAAGGTGAAGGTCGGAGTC -3’
**GAPDH-R**	5’- GAAGATGGTGATGGGATTTC -3’

F: forward primer; R: reverse primer.

### Western blot

Total proteins of the hepatoma carcinoma cells were firstly extracted by RIPA Lysis buffer (Beyotime, Beijing, China), and then quantified by BCA kit (BioTeke, Beijing, China). Separated by sodium dodecyl sulfate-polyacrylamide gel electrophoresis (SDS-PAGE), the cell lysate (25-100 μg of protein) was electrophoretically transferred onto polyvinylidene fluoride (PVDF) membrane (Thermo Scientific™, Waltham, MA, USA). Blocked with 5% non-fat milk, the membrane was incubated with dilute primary antibodies overnight at 4˚C. After being rinsed with PBS 3 times, the membranes were incubated with dilute secondary antibodies for 1 h at room temperature. After incubation, the membranes were washed with PBS. The PVDF membranes were detected with enhanced chemiluminescence (ECL) reagent (Cell Signaling Technology, Danvers, MA, USA) in darkroom after exposure and development. The primary antibodies and the secondary antibodies were listed in Table 2. The LC3B antibody we used could identify the totally endogenous LC3B, including both LC3B-I and LC3B-II, whose molecular weights were 16kD and 14kD, respectively. It could detect the transformation from endogenous LC3B-I to LC3B-II.

**Table 2:** Primary antibodies and secondary antibodies.

**Proteins**	**Antibodies**
**BCL2L10-P**	Anti-BCL2L10 antibody (1:1000, Abcam, # ab96625)
**BCL2L10-S**	Goat Anti-Rabbit IgG H&L (HRP) (1:2000, Abcam, #ab205718)
**LC3B-P**	Anti-LC3B antibody (1:1000, SIGMA)
**LC3B-S**	Goat Anti-Rabbit IgG H&L (HRP) (1:2000, SIGMA)
**phospho-Akt-P**	Anti-AKT1 (phospho S473) antibody (1:10000, Abcam, # ab81283)
**phospho-Akt-S**	Goat Anti-Rabbit IgG H&L (HRP) (1:2000, Abcam, #ab205718)
**PI3K p110α-P**	PI3 Kinase p110α Antibody (1:1000, Cell Signaling, #4255)
**PI3K p110α-S**	Anti-rabbit IgG, HRP-linked Antibody (1:2000, Cell Signaling, #7074)
**Akt-P**	Akt Antibody (1:1000, Cell Signaling, #9272)
**Akt-S**	Anti-rabbit IgG, HRP-linked Antibody (1:2000, Cell Signaling, #7074)
**P62-P**	Anti-p62 antibody (1:1000, Abcam, # ab56416)
**P62-S** **Beclin 1-P** **Beclin 1-S** **β-actin-P** **β-actin-S** **Myc** **BECN1**	Goat Anti-Mouse IgG H&L (HRP) (1:2000, Abcam, #ab205719) Anti-Beclin 1 antibody (1:1000, Abcam, #ab62557) Goat Anti-Rabbit IgG H&L (HRP) (1:2000, Abcam, #ab205718) Anti-beta Actin antibody (1:500, Abcam, #ab8226) Goat Anti-Mouse IgG H&L (HRP) (1:2000, Abcam, #ab205719) Anti-Myc antibody (5 µg/ml, Abcam, ab32072) Anti-BECN1 antibody (1:30, Abcam, ab207612)

P: Primary antibodies; S: Secondary antibodies.

### Immunohistochemistry

After deparaffinization and rehydration using xylene and ethanol, sections (4 mm thick) from paraffin-embedded tumor tissues were immersed for 10 min in 3% hydrogen peroxide solution (Sigma-Aldrich, St. Louis, MO, USA). After rinsed with PBS, sections were added with primary antibodies (1:500), secondary antibodies (1:2000), streptavidin-peroxidase solution (S-P, Thermo fisher), and diaminoaniline (DAB, # ab64261, Abcam, Cambridge, MA, USA) for 3-5 minutes. Counterstained with hematoxylin (Beyotime, Jiangsu, Shanghai), sections were observed under an optical microscope (Olympus, Tokyo, Japan).

### CCK-8 assay

48 hours after the transfection, HCC cells were digested, centrifuged and made into cell suspension with serum-supplemented medium. The cells were cultured at 37 °C with 5% CO_2_ for 12 h, 24 h, 36 h, 48 h, 60 h and 72 h respectively in 96-well plates (100 μL/8,000 cells per well). Then, CCK-8 kit (Dojindo, Kumamoto, Japan) (10 μL/well) was added. After incubation for 4 h, optical density (OD) value of the cells was measured using the Thermo Scientific Microplate Reader at 450 nm.

### Drug preparation and Bafilomycin A1 treatment

LY294002 (#9901), MK2206 (#S1078) and 3-methyladenine were purchased from Cell Signaling Technology, Selleck Chemicals and Sigma-Aldrich, respectively. HCC cells were incubated in complete medium supplemented with 3-methyladenine and PI3K inhibitor (LY294002) and AKT inhibitor (MK2206) for the related assays. Bafilomycin A1 was diluted in dimethylsulfoxide (DMSO) to a final working concentration of 1 µmol/L and DMSO was used as the vehicle control. Cells treated with Bafilomycin A1 for 24 h after cell transfection were harvested and washed three times with 5 ml PBS/0.1% fetal calf serum wash buffer, centrifuged and aspirated. The cells were then prepared for the follow-on experiments.

### Immunofluorescence staining of *BCL2L10* and Beclin 1 and confocal microscopy

Immunofluorescence assay were performed to show that *BCL2L10* and Beclin 1 were co-located in hepatoma cells. Cells were collected, fixed and permeabilized with 0.3% triton X-100 for 10 min, and then incubated with first primary antibody to BCL2L10 (1:200) at 4ºC overnight. Fluorescein (FITC) conjugated Goat Anti-Rabbit IgG (1:400) was applied for 1.5h at room temperature. Then hepatoma cells were incubated with the second primary antibody, rabbit anti- Beclin 1 (1:200) at 4ºC overnight. Rhodamine (TRITC) conjugated Goat Anti-Rabbit IgG (1:400) were finally applied for 1.5h. Blocking solution for immunofluorescence was 10% NSS. Cell nuclei were stained using ProLong Gold Antifade Reagent with DAPI (Life Technologies Corporation, Gaithersburg, MD, USA). Samples were examined under an FLUOVIEW FV1000 confocal laser scanning microscope (Olympus). The excitation/emission wavelengths for Rhodamine (TRITC) and Fluorescein (FITC) were 490/516 and 577/590 nm, respectively.

### MRFP-GFP-LC3 Autophagy assays

As mentioned above, cells were transfected with si-*BCL2L10*/ si-*BECN1* (transformed into shRNAs and sub-cloned into pcDNA3.1 vectors), pcDNA3.1-*BCL2L10*/pcDNA3.1-*BECN1* plasmids, pTRE/BCL2L10ΔBH1, PI3K inhibitor (LY294002), or AKT inhibitor (MK2206). Months later, stably resistant cells with the antibiotic resistant marker were screened to collect the stable-transfected cell lines. To analyze autophagic flux, we monitored the formation of autophagic vesicles by the dual- fluorescence mRFP-GFP-LC3 method (Invitrogen). Due to the quenching of GFP in the acidic lysosomal environment, we could distinguish the autophagosomes and autolysosomes through detecting both mRFP and GFP signals, followed by only the mRFP signal. Stable-transfected Hep3B cells were transfected with mRFP-GFP-LC3 expressing pLenti6 lentivirus (Nanjing Mergene Life Science, Nanjing, China). Autophagic flux was determined by evaluating the punctuated pattern of GFP and mRFP. 24 h after transfection, cells were then transfected with other plasmids or siRNAs (using Lipofectamine 3000), washed with PBS and fixed with 4% paraformaldehyde. The GFP and mRFP puntas were observed with a 60×objective lens. 4’, 6-diamidino-2-phenylindole (DAPI) was used to stain cell nuclei under permeabilizing conditions. Fluorescence was analyzed on the confocal laser scanning microscope using Cell M software (FV10i, Olympus, Tokyo, Japan). The protein levels of LC3B and P62 were determined by Western blotting.

### Co-immunoprecipitation

After transfection, cells were collected and lysed by frozen EBC lysis buffer (Invitrogen). After centrifugation of 10 μl precleared cell lysate for 15 min, protein concentration in the supernatant was determined by bicinchoninic acid assay (BCA). 30μg Protein A or protein G agarose/sepharose (Millipore, Billerica, MA, USA), anti-flag antibody (SIGMA) or anti-Myc antibody (Abcam, Cambridge, MA, USA) were added to the supernatants at 4˚C, which was subsequently incubated with various irrelevant immunoglobulin-G (IgG) or anti-BECN1 antibody (Abcam) in the presence of protein A or G agarose/sepharose beads for 2 h or overnight at 4°C with gently shaking. Following incubation, agarose/sepharose beads were collected and washed 5 times with lysis buffer. Then, the complex was eluted at 100˚C for 4 min. The eluate was collected and subjected to SDS-PAGE for Western blot analysis. Antibodies were listed in Table 2.

### ELISA

Cells from different cell groups were treated as required and cells were collected and split according to the instruction of PI3K ELISA Pico Assay kit (Lanpaibio, Shanghai, China). The lysed cellular protein was incubated with PI3KC3 antibody overnight at 4 °C. Then, the lysed cellular protein was added with Protein A/G agar and incubated for 3 hours at 4 °C. After centrifugation, the lysed cellular protein was washed 3 times. The PI3KC3 kinase was dissociated using the protein dissociation solution in the kit for subsequent activity detection. The specific procedure was followed by the kit instructions.

### Statistical approach

Each in vitro experiment was conducted for three times to obtain the average value. The image of immunofluorescence was collected by Sigma Pro 5 software (Sigma-Aldrich, St. Louis, MO, USA). Graph Pad Prism 6 software was adopted for analysis of the experimental results. Results were expressed as the mean ± standard (SD) error of the mean with at least five independent experiments. For multiple comparisons, One-way analysis of variance (ANOVA) was employed. Two-group comparisons were conducted using Student’s t-test. *P*<0.05 indicated statistically significant differences.

### Ethics approval and consent to participate

All procedures followed were in accordance with the ethical standards of Guangdong General Hospital, Guangdong Academy of Medical Sciences, and obtained written informed consents from all the participants.

## Supplementary Material

Supplementary Figures

Supplementary Tables
