# Supplementary material for: BCL2L10/BECN1 modulates hepatoma cells autophagy by regulating PI3K/AKT signaling pathway
Source: Aging (Albany NY). 2019 Jan 26;11(2):350–70. doi: 10.18632/aging.101737 (PMC6366968; doi:10.18632/aging.101737)
Supplement: Supplementary Figures [file aging-11-101737-s001.pdf]

SUPPLEMENTARY FIGURES

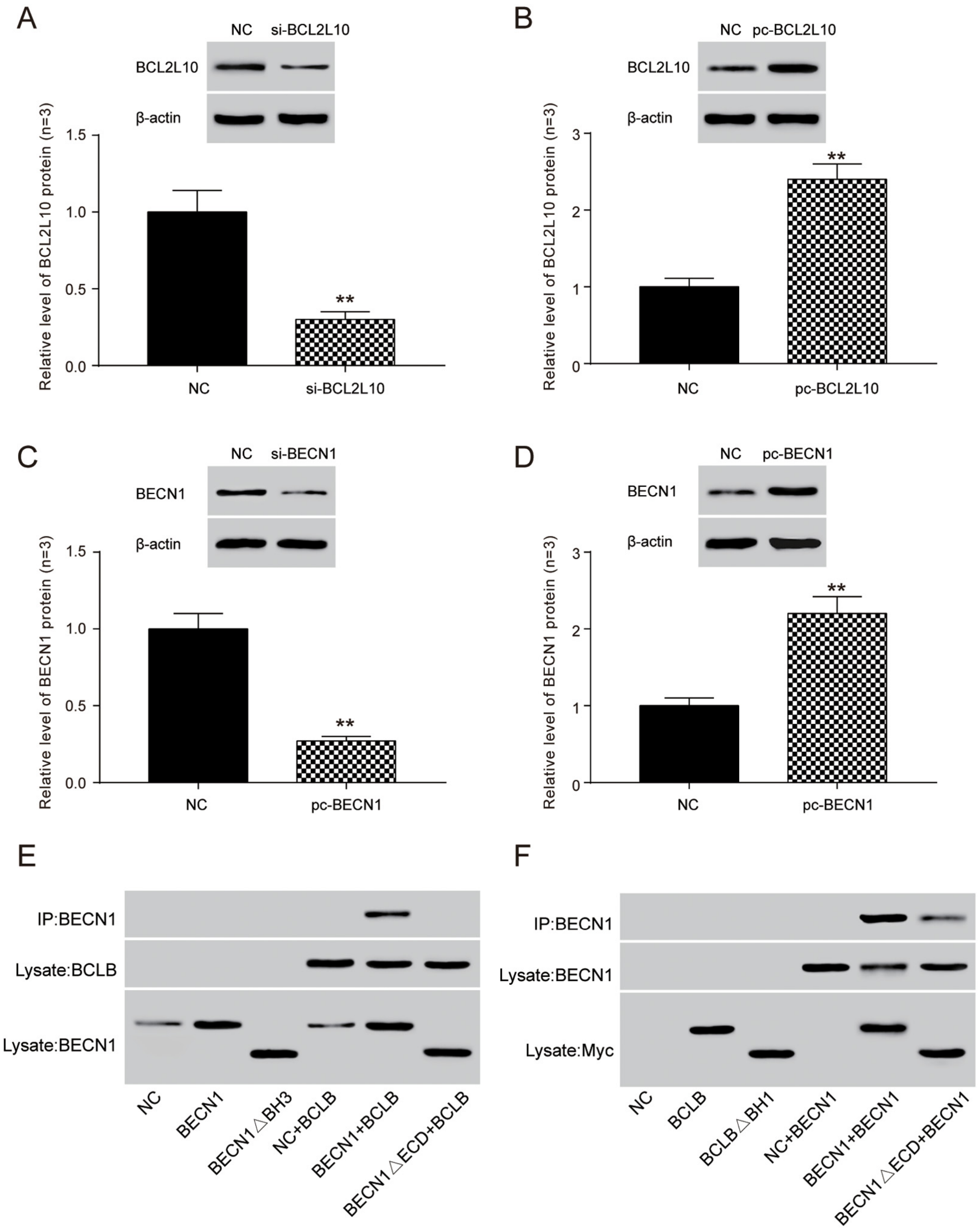

Supplementary Figure 1. The BECN1 BH3 domain and BCL2L10 domain BH1 were required for BCL2L10-BECN1 complex in Hep3B cell line. The protein levels of BCL2L10 (**A**-**B**) and BECN1 (**C**-**D**) in OE/KD groups were detected by Western blot to verify the transfection efficiency. (E-F) The binding sites on each protein were indicated by Co-IP with BCL2L10 antibody.

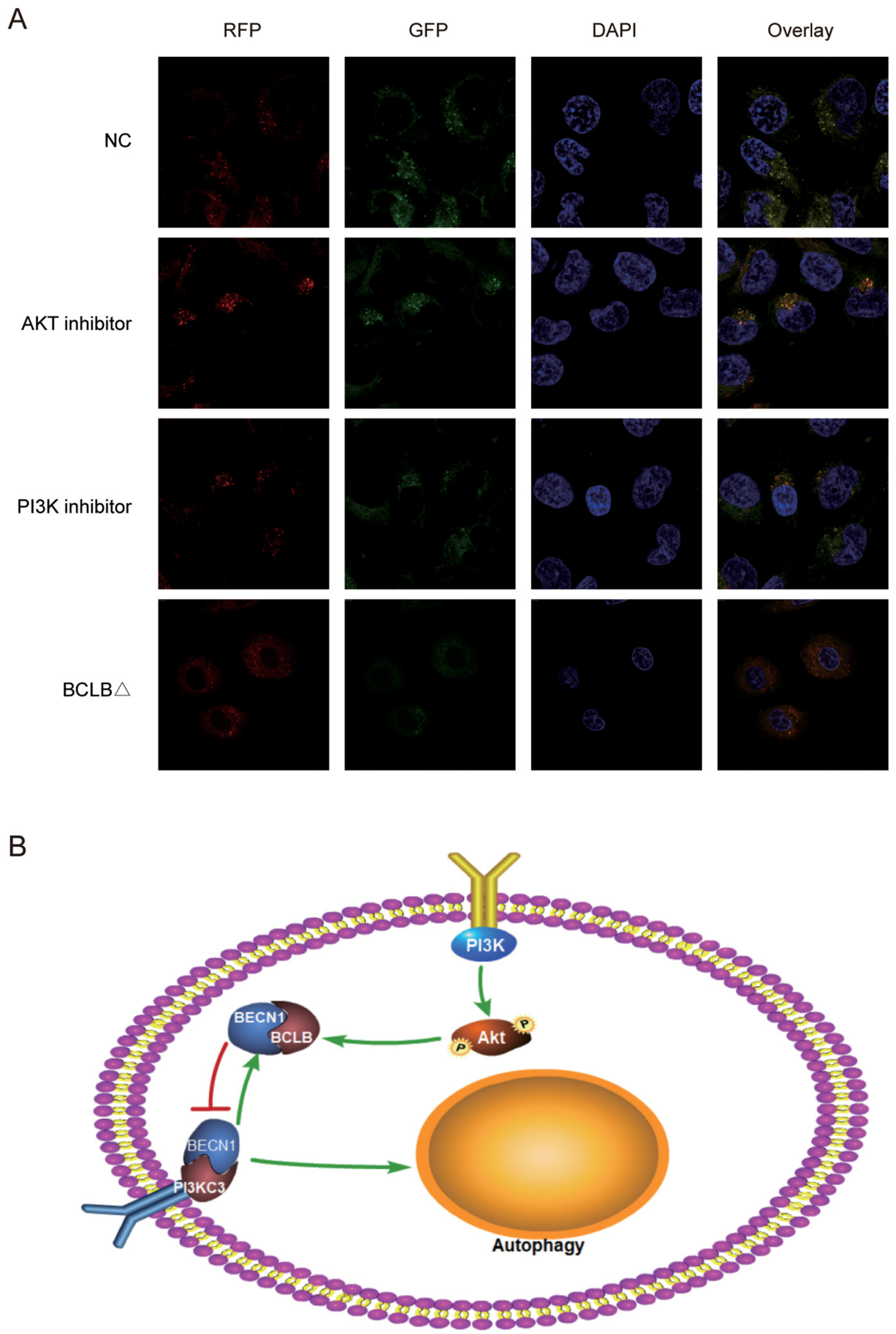

Supplementary Figure 2. A model depicting a novel mechanism by which BECN1 induces Hep3B cell autophagy. (**A**) Mutant the BH1 domain on Beclin1 or the inhibitor of PI3K/AKT signal could induce autophagic flux in Hep3B cells according to mRFP-GFP-LC3 assay. (**B**) A molecular mechanism diagram describing BCL2L10 competitive binding BECN1 inhibiting the BECN1-mediated autophagy.
