# Supplementary material for: BCL2L10/BECN1 modulates hepatoma cells autophagy by regulating PI3K/AKT signaling pathway
Source: Aging (Albany NY). 2019 Jan 26;11(2):350–70. doi: 10.18632/aging.101737 (PMC6366968; doi:10.18632/aging.101737)
Supplement: Supplementary Tables [file aging-11-101737-s002.pdf]

SUPPLEMENTARY TABLES

Supplementary Table 1. Clinicopathologic information of hepatoma carcinoma (HCC) patients.

Parameters	Group	HCC (n=50)
Gender	Male	28
	Female	22
Age	< 60	19
	≥ 60	31
Stage	□	20
	□	11
	□	18
	□	1

Supplementary Table 2. Clinical characteristics of participants in GSE49515.

Parameters	Group	HCC (n=114)
Gender	Male	103
	Female	11
Average age	56 (29-83)	114
Alpha-fetoprotein (AFP)	>20 ng/ml	71
	<20 ng/ml	43
Tumor size	<3 cm	28
	3-5 cm	27
	>5 cm	55
	Not available	4
Barcelona-Clinic Liver Cancer (BCLC) stage	Stage 0	13
	Stage A	30
	Stage B	32
	Stage C	21
	Stage D	13
	Not available	5
TNM stage	T1	34
	T2	21
	T3	50
	T4	4
	Not available	5
